# Challenges in Glioblastoma Radiomics and the Path to Clinical Implementation

**DOI:** 10.3390/cancers14163897

**Published:** 2022-08-12

**Authors:** Philip Martin, Lois Holloway, Peter Metcalfe, Eng-Siew Koh, Caterina Brighi

**Affiliations:** 1 Centre for Medical and Radiation Physics, School of Physics, University of Wollongong, Wollongong, NSW 2522, Australia; 2 Ingham Institute for Applied Medical Research, Liverpool, NSW 2170, Australia; 3 Liverpool and Macarthur Cancer Therapy Centres, Liverpool, NSW 2170, Australia; 4 South Western Sydney Clinical Campus, School of Medicine, University of New South Wales, Liverpool, NSW 2170, Australia; 5 ACRF Image X Institute, Sydney School of Health Sciences, Faculty of Medicine and Health, The University of Sydney, Sydney, NSW 2006, Australia

**Keywords:** radiomics, Glioblastoma, machine learning, MRI, biomarker

## Abstract

**Simple Summary:**

Glioblastoma (GBM) is a cancer with poor prognosis, its 5-year survival expectation is approximately 5%. Advances in oncologic treatment techniques have not led to significant improvements in survival outcomes for GBM patients. Part of the reason for the treatment failures in GBM patients is that treatments fail to account for heterogeneities both within and between different tumors. Radiomics is a rapidly emerging research field that examines the relationship between medical imaging features and patient clinical outcomes and biological characteristics of tumours. This review outlines the applications of radiomics for GBM patient management and the barriers facing the implementation of radiomics into clinical practice. In completing this review, we hope to inform clinicians and researchers on how radiomics may be used to improve patient clinical outcomes.

**Abstract:**

Radiomics is a field of medical imaging analysis that focuses on the extraction of many quantitative imaging features related to shape, intensity and texture. These features are incorporated into models designed to predict important clinical or biological endpoints for patients. Attention for radiomics research has recently grown dramatically due to the increased use of imaging and the availability of large, publicly available imaging datasets. Glioblastoma multiforme (GBM) patients stand to benefit from this emerging research field as radiomics has the potential to assess the biological heterogeneity of the tumour, which contributes significantly to the inefficacy of current standard of care therapy. Radiomics models still require further development before they are implemented clinically in GBM patient management. Challenges relating to the standardisation of the radiomics process and the validation of radiomic models impede the progress of research towards clinical implementation. In this manuscript, we review the current state of radiomics in GBM, and we highlight the barriers to clinical implementation and discuss future validation studies needed to advance radiomics models towards clinical application.

## 1. Introduction

Glioblastoma multiforme (GBM) is the most common primary brain malignancy with an incidence of 3.1 cases per 100,000 adults in the United States [1]. Despite a multimodal treatment regime of maximal safe resection [2] and adjuvant chemoradiation therapy, the prognosis is poor for GBM patients. Median overall survival is approximately 15 months, and the 5-year survival rate is 5.1% [3].

The current standard of care for GBM consists of maximal safe resection followed by concurrent Temozolomide (TMZ, 75 mg/m^2^/day) and radiation therapy (60 Gy over 30 fractions delivered over six weeks). In elderly patients, it has been found that there is no significant difference in outcomes between patients treated with hypofractionated radiotherapy (40 Gy in 15 fractions over three weeks) and conventionally fractionated radiotherapy [4,5]. Recent developments in treatment include the use of low intensity, intermediate frequency electric fields. These Tumour Treating Fields (TTFs) have been found to arrest cell division and cause death in tumour cells [4]. Prognosis is limited by tumour progression, which occurs in over 80% of GBM patients, demonstrating the inefficacy of current treatment methods. Multiple factors have been cited as contributing to treatment failure [6]. These factors include intra-tumoural heterogeneity, referring to the physiological variations between different tumour regions, and inter-tumoural heterogeneity, referring to the physiological differences between tumours from different patients. This heterogeneity contributes to common treatment failure as homogeneous treatment prescriptions are ineffective in GBM treatment [7]. Treatment failures in GBM can be attributed to the tumour’s ability to dynamically adapt and develop mechanisms of treatment resistance. Resistance to chemotherapy arises from the presences of the blood–brain barrier (BBB) preventing chemotherapy drugs from reaching tumour cells and from inherent molecular resistance to these drugs [6]. Resistance to radiotherapy arises from tumour hypoxia reducing the treatment efficacy [8]. Additionally, peritumoural infiltration of tumour cells in the brain parenchyma is another important hallmark of GBM contributing to treatment failure and local relapse [9].

Magnetic Resonance Imaging (MRI) is the primary imaging technique used for the diagnosis, treatment planning and monitoring of GBM [10]. This is due to the excellent soft tissue contrast offered by MRI and the ability to change acquisition parameters to enhance contrast between normal and diseased tissue. Anatomical MRI sequences routinely acquired for the clinical management of GBM include T1-weighted (T1-w), T1-weighted contrast enhanced (T1CE), T2-weighted (T2-w) and T2 fluid attenuated inversion recovery (T2-FLAIR) imaging [11]. Gadolinium-based contrast agents, which are administered during T1CE imaging acquisition, enable improved delineation of the tumour boundaries. However, as selective accumulation in the tumour tissue relies on the extravasation of the contrast agent into the extracellular extravascular space, this technique is only useful for the delineated tumour regions with a disrupted BBB [9], leaving infiltrating tumour regions with an intact BBB invisible. The contrast-enhancing tumour volume delineated from T1CE imaging is normally used to define the gross tumour volume for planning radiotherapy. As such, this volume is often an underestimation of the ‘entire tumour burden’ [12]. FLAIR imaging is also utilised in delineating GBM tumour volumes on MRI. Hyperintense T2-FLAIR volumes are used in the delineation of tumour radiotherapy volumes, especially in defining areas of peritumoural cancer infiltration in oedema [13]; however, this has limitations as the prognostic value of FLAIR signal has not been clearly shown in GBM tumour response assessment [13]. Radiomics could help provide a method to quantify the extent of tumour infiltration beyond the contrast enhancing boundaries [12,14], potentially enabling the adaptation of treatment plans tailored to the characteristics of each patient’s individual tumour [15]. Functional imaging approaches such as quantitative MRI (qMRI) and Positron Emission Tomography (PET) have also been increasingly utilised in the management of GBM as they can provide information about the tumour physiological processes, including vascularisation, cellularity, hypoxia and metabolism [10,16].

Radiomics is a method of data analysis in which many quantitative features are extracted, and machine learning (ML) methods are used to establish correlations with patient clinical outcomes [17,18]. The underlying hypothesis of radiomics is that radiomic features reflect the biological characteristics of the tissue, which in turn are related to patient clinical outcomes. While previous studies have demonstrated that radiomics have the potential to predict clinical outcomes in GBM [19,20,21,22,23], its application is still far from clinical implementation [24,25]. Radiomics models for GBM are generally based on MRI due to its central role in the management of brain cancer patient; however, radiomics approaches using CT and PET imaging have also been investigated [26,27].

Radiomic models can be assessed based on three key factors: performance is defined by the accuracy of the model’s predictions; clinical utility determines whether the predictions can be used clinically to modify treatment and improve patient outcomes and transferability, which represents the ability of the model to maintain performance when applied to datasets with varied acquisition parameters [24,28].

The progress of radiomics in GBM is reviewed by outlining the radiomics methodology as a whole and how this may be improved, performing a review of the existing models in GBM radiomics and outlining what improvements are necessary to build confidence in radiomics models. The current state of radiomics research in GBM, the gaps in current research, specific challenges in developing a radiomics model for brain cancer and how models may build confidence to advance to a state of clinical implementation are outlined. This knowledge is essential for both clinicians and data experts to advance radiomics research in GBM.

## 2. Literature Selection Methodology

In this review, we selected studies that use innovative techniques such as ML and quantitative MRI for the development of radiomic models. The performance and potential clinical utility of radiomics models developed using these methods is likely to be critical to the continued progress and clinical implementation of radiomics in GBM patient management. Particular attention has been paid to studies that advances the state of research towards clinical implementation. Studies for this review were found by searching the PubMed Central database with keywords of ‘GBM’, ‘radiomics’, ‘machine learning’ and ‘quantitative MRI’. Studies published since 2016 and important papers in the history of radiomics have been included in this review. Particular attention has been paid to studies that have used innovative methods such as ML or multi-parametric imaging.

Literature was selected in this review to outline the progress of radiomics in GBM, including the development and implementation of novel methods within the radiomics pipeline. By reviewing literature that implemented these novel methods, we hope to investigate how radiomics models may build confidence by improving their performance, clinical utility and transferability.

## 3. Radiomics

### 3.1. Radiomics Methodology

**Figure 1:**
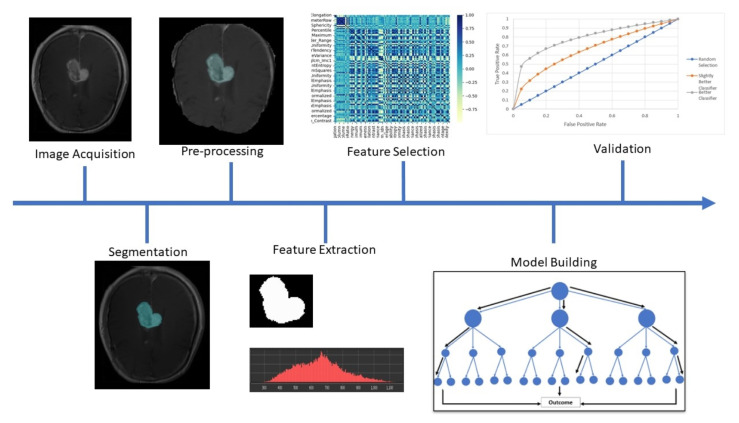
A scheme representing the process of developing a radiomics model. (1) Image Acquisition, (2) Tumour Segmentation, (3) Image Pre-processing and standardisation, (4) Feature Selection, (5) Statistical Model Building and (6) Statistical Validation.

The process of developing a radiomics model involves the steps of image acquisition, pre-processing, segmentation, feature extraction, model building and validation [29], which are represented in Figure 1.

Image acquisition involves the collection of images of the tumour and surrounding tissue. MR images are heavily affected by acquisition parameters including pulse sequence, pixel size and scanner specifications. The variation in acquisition parameters between different centres limits the transferability of radiomics models [30].

Segmentation involves outlining tumours and other regions of interest on the acquired images. Consistent segmentation is critical to the development of radiomic models. Segmentation is typically performed by radiation oncologists; however, ML algorithms have also been used for this task [11]. Inter-rater variability, which refers to the variation between the segmentations produced by different human raters, has been identified as a factor that can reduce the robustness of radiomic features [31].

Image pre-processing is the process of performing image transformations to standardise similar images acquired with different protocols. Whilst not strictly necessary, pre-processing should be implemented for models incorporating datasets with varied acquisition parameters. Common algorithms used in standardisation of brain MRI include pixel resampling (usually to a common pixel size of 1 mm^3^), intensity standardisation and bias correction [32].

Feature extraction involves the calculation of radiomic features from regions of interest. Radiomic features are classified into one of four categories; shape, first-order, second-order and higher order. The calculation and utility of these features will be discussed in further detail in Section 3.2.

Feature selection and model building consist of identifying correlations between features and relevant biological or clinical parameters. To construct a viable radiomics model from the large number of radiomics features, it is necessary to select features representative of clinical or biological parameters of interest. Features with high correlation to each other (redundant features) must first be eliminated from a model [17]. From the remaining features, those with the highest correlation to relevant outcomes are incorporated into the model. These features can either be correlated to the outcome through statistical methods (e.g., regression) or through ML algorithms (e.g., Random Forest, Neural Network, Support vector machine) [33].

Model validation consists of testing the model on unseen data and measuring the accuracy of predictions made on this data. There are multiple methods that are used to assess the performance of a radiomics model. Validation usually requires two distinct datasets, one for training and one for testing [34]. Ideally, an external dataset is used for testing so that a more realistic estimate of the performance and generalizability of the model can be made. However, most studies only use single datasets without external testing datasets [35]. Cross validation is another method that is useful for small datasets. This involves splitting a dataset equally into k subsamples. One subsample is used for validation, and the remaining subsamples are used for training. This is repeated k times [34] with each subsample used once for validation. The results are then obtained by averaging the performance of each of the validation steps.

Variations at any point within this process can result in a reduction in accuracy or reliability of radiomic models [32,36].

### 3.2. Radiomic Features

Taken as a whole, radiomics features can be thought of as ‘summary statistics’ for a region of interest on an image. That is, that they evaluate certain characteristics of an image using a single number.

**Shape features**: Shape features describe the geometric properties of the region of interest, such as volume or surface area.

**First order features**: First order features relate to the pixel values of a region of interest and can be derived from a histogram of these pixel values. Features of this type include the mean, skewness, standard deviation and energy of the pixel values.

**Second order features**: Second order features relate to the spatial distribution of pixel values and are extracted from a set of matrices that describe the texture of images. These features are calculated using a set of matrices that are related to the spatial distribution of pixel values. Matrices used for this purpose include the Gray-Level Co-occurence Matrix (GLCM), which considers the number of times a pixel intensity occurs along a certain axis, and Gray-Level Run Length Matrix (GLRLM) which quantifies the length of segments with the same pixel intensity.

**Higher Order features**: Higher order features are first or second order features that are extracted from an image after image transformations, such as filtering or wavelet transformations, have been applied [37].

Feature extraction in most radiomics studies has been limited to the traditional ‘handcrafted’ features, being features with a predefined mathematical definition. Deep or learned features are another type of feature that have received attention in recent years [23,38,39]. These are features that are derived using convolutional neural networks. Rather than being explicitly defined mathematically, the neural network used to derive these has been ‘trained’ to produce a prediction of a certain outcome.

Software such as MATLAB and Python is often used to extract radiomic features. Open source packages for the calculation of radiomics features are common, including PyRadiomics for Python, and Computational Environment for Radiotherapy Research (CERR) for MATLAB [40]. Some variation in radiomic features extracted by different software packages [41] has been found so it is important for studies to standardise their feature extraction workflows for transferability and comparability.

### 3.3. Artificial Intelligence and Radiomics

Artificial intelligence (AI) methods and radiomics have a natural synergy. AI describes a group of computational algorithms that can form predictions based on a large amount of data [33]. Increasingly large datasets used to create radiomic models can benefit from AI algorithms in generating accurate predictions. In particular ML, a group of algorithms which improve the accuracy of their predictions by performing analysis on unseen data shows promise in medical imaging analysis.

ML algorithms are generally classified as supervised, where data are accompanied by relevant labels that a model ‘learns’ to predict, or unsupervised, where no labels are provided with the data and the algorithm attempts to discern patterns within the data. Many ML algorithms, including support vector machines, neural networks and random forest, have found utility in radiomics [42]. Neural networks are a set of ML methods where data are fed through a set of interconnected ‘layers’ of linear operations to produce predictions. Support vector machine is an ML algorithm that aims to perform binary classification on multi-variate data by identifying the best classification threshold based on multi-dimensional data. Included in Table 1 below is a summary of some ML methods used in conjunction with radiomics [15,33].

ML methods can be integrated into the radiomics pipeline at multiple points [12,22,23]. Segmentation has been a highly investigated ML-based task in GBM research, due to the wealth of data available expressly for this purpose [11]. ML methods also have the potential to supplement or replace the linear regression traditionally used in building a radiomics model [12,21] and have shown to provide improved performance [12,22].

### 3.4. Implementation of Standardisation Methods

MRI signal is derived from a complex interplay of tissue properties and scanner parameters. As a result, significant variability in the accuracy of radiomics models can arise from variation in acquisition parameters or from feature instability. Effective model building requires the features extracted from medical images to be repeatable and reproducible. Efforts to standardise imaging practice have been recommended by the Quantitative Imaging Biomarkers Alliance (QIBA) [48,49]. These guidelines recommend quantifying repeatability, a measure of biomarker variability over a short time when all acquisition and processing parameters are kept constant; and reproducibility, where acquisition or processing parameters are altered in some way [50]. Additionally, specific recommendations for acquisition parameters in diffusion-weighted and dynamic contrast enhanced MRI have been suggested to improve precision of imaging biomarkers derived from these sequences [49].

Methods to improve repeatability and reproducibility in radiomics include intensity standardisation, volume resampling, bias field correction and noise filtering [51,52]. Intensity standardisation is a method used to reduce variability in grey-level between images. This is particularly important for MRI, where pixel values do not necessarily represent a physical quantity and can thus vary depending on scanner model and acquisition parameters. Methods used for intensity standardisation include mapping all pixel intensities to a specific range, e.g., [0, 255], z-score, histogram matching or Gaussian normalisation [37,53]. Volume resampling involves adjusting the spatial resolution to a common pixel size, usually 1 mm^3^, and cropping the image to a size of 256^3^ voxels. Bias field correction is a method used to eliminate bias field signal, a low frequency smooth signal produced by inhomogeneity in the radiofrequency field of MRI. Multiple methods have been suggested to eliminate this signal [37,54]. A study by Hoebel et al. [32] implemented a selection of the above standardisation methods to determine their effect on the repeatability of radiomic features extracted from structural MRI by way of test–retest imaging. In this study, repeatability was measured by comparing features between the two scans. A similar study by Suter et al. [36] assessed the repeatability of features under various image perturbations. Studies of this nature allow non-repeatable and/or non-reproducible features to be eliminated from a predictive model, thus contributing to a better model transferability [17].

Implementation of these standardisation methods can improve comparability of models developed at different centres [36]. However, it should be noted that these techniques may also increase the correlation between different features [32,51]. High correlation between features may result in reduced model accuracy, as information is lost in pre-processing. Therefore, it is recommended that the effect of pre-processing methods that improves the repeatability of radiomic features of interest is validated in studies utilising data with diverse acquisition protocols.

## 4. Current State of Research in GBM Radiomics

Radiomics models have performed well predicting clinical and biological characteristics of tumours. The performance of these models has continued to improve as more data have become publicly available and innovative artificial intelligence methods have been utilised in radiomic analysis.

### 4.1. Potential Applications of Radiomics in GBM Patient Management

To date, clinical assessments of patient status have been primarily based on qualitative metrics [37]. This presents two main issues. First, basing clinical decisions on qualitative metrics introduces observer bias to treatment. Second, the qualitative metrics used are not sufficient to describe the functional characteristics of the tumour. Radiomics could help to address these issues by providing a quantitative, unbiased method of assessing tumour physiology [37].

Tasks in the management of GBM patients that radiomics are suited for including diagnosis, prognostication, stratification, response assessment and treatment planning.

In the context of diagnosis, radiomics could reduce the need for invasive biopsies through the automatic detection and grading of brain tumours [47,55]. The ‘gold standard’ for the grading of gliomas is biopsy [56]. Interestingly, a recent study by Kobayashi et al. [39] was able to achieve an accuracy of 0.90 ± 0.03 using a fully automated radiomics model for distinguishing between high and low grade glioma. This was based on structural MRI sequences from the Brain Tumour Segmentation (BraTS) challenge [11,45,57]. The use of radiomics for these purposes could reduce the need for invasive surgical procedures as well as reducing observer bias.

Prognostication predicts patient clinical outcomes and can be completed to identify if a patient would benefit from a more aggressive treatment regimen. Studies attempting to predict patient overall survival have generally performed well when incorporating advanced methods such as ML analysis or when incorporating multi-parametric imaging [19]. A prognostic task that radiomics could be useful for in GBM patient management is the identification of O-6-methylguanine-DNA methyltransferase (MGMT) promoter methylation status. MGMT is a repair protein which inversely correlates to patient survival [58]. MGMT methylation is both a prognostic and predictive marker, conferring an improved survival and better response to TMZ treatment [59]. Studies aiming to determine the MGMT methylation status [60,61] have seen some success using radiomic features extracted from structural MRI sequences. Improvements on these predictions and treatment implementation could help clinicians identify patients that may benefit from TMZ chemotherapy.

Stratification classifies patients based on important clinical factors and can help determine if a patient may benefit from certain treatments. This is often completed in conjunction with prognostication. For example, a study by Kickingereder et al. [62] demonstrated the utility of radiomics in identifying patients who may benefit from anti-angiogenic therapies. This study was able to achieve an area under the receiver operator curve (AUC) of 0.792 for prediction of overall survival for patients treated with bevacizumab.

Treatment response assessments in GBM clinical trials often rely on the updated Response Assessment in Neuro-Oncology (RANO) criteria [63]. These are based on changes in tumour volume and contrast enhancement observed on T1CE images. Radiomics could also be used to measure functional tumour changes occurring over time in response to treatment [14]. The ability of radiomics to quantify underlying tissue physiology makes it suitable for assessing tumour treatment response [18]. Diffusion-weighted imaging has been investigated as a potential predictor of early treatment response for GBM [64]. Recent studies implementing radiomics have shown to be able to differentiate between true tumour progression and pseudo-progression with a sensitivity of approximately 80% [20,27]. The combination of qMRI sequences and radiomics analyses has the potential to generate predictive models of tumour response, which could help clinicians make timely decisions for treatment adaptation [20].

Treatment planning is another promising application of radiomics in GBM management. For instance, radiotherapy stands to benefit from the integration of radiomics into clinical practice [15,65,66]. Radiomics may assist in the segmentation of radiotherapy target volumes and in measuring and predicting treatment response [67]. Identification of peritumoural infiltration is also a task which can improve the quality of surgery and radiotherapy by providing a means to better define tumour margins, and is well suited to be performed by radiomics [12,66]. Tumour hypoxia is a factor which reduces the efficacy of radiotherapy as DNA damage is reduced in hypoxic regions. Radiomics has shown potential in the identification of hypoxic tumour regions [8], which could be leveraged for dose escalation treatment strategies.

### 4.2. Existing Models in GBM Radiomics

To date, the best performing models have incorporated functional imaging and/or ML methods to improve predictions. A selection of radiomics models in GBM has been outlined below. These models have been selected as they address one of the key factors for the progress of radiomics towards clinical implementation (being clinical utility, performance and transferability). Table 2 reports recent studies with a focus on radiomics in GBM.

These models highlight how radiomics can build confidence to progress towards clinical implementation. Each model has its own clinical utility. Survival prediction models [19,23,38] can be used to determine if a patient may benefit from a more aggressive treatment regimen, the prediction of peritumoural infiltration [12] and the prediction of local vs. distant recurrence [22] could allow for improved treatment planning and stratification studies [62] can help determine if a patient can benefit from a certain treatment regimen. Innovative methods such as deep learning and mpMRI were implemented in these models to improve their performance. Suter et al. [36] investigated the repeatability of radiomic features and the performance of a model when applied to images acquired with different acquisition protocols to the training dataset. This study was an excellent example of a method that could be used to measure and improve transferability of radiomics models.

One problem hindering the clinical translation of radiomic models is the lack of multi-centre data being utilised for training and technical validation. By training and validating a model on single-centre data, the issue of inter-centre variability is largely neglected. Inter-centre variability refers to variability in image intensity due to differences in scanner or acquisition protocols, which has been found to have a significant impact on MRI and MRI-derived radiomic features [51,68]. Technical validation studies aim to investigate the performance of models when applied to data not drawn from the same dataset used for training [69]. Some of these studies have been completed to date [25,36]; however, further studies will be required so that reproducibility of model results can be assured.

### 4.3. Challenges of Developing a Radiomics Model for Brain Cancer

For any model to reach clinical implementation, it needs to fulfill several criteria. It should provide valuable information that can improve the clinical workflow; it should produce accurate predictions of underlying physiology/clinical outcomes (clinical/biological validation); it should yield reproducible predictions on images acquired from different centres (technical validation); and it should be statistically sound, meaning that it has been trained on a large amount of diverse data [37]. A greater number of patients in the training cohort produces a more accurate model; thus, there is no upper limit on the number of patients required for the development of a radiomics model. As a guideline for a statistically robust dataset, there should be at least 10 patients per radiomic feature implemented in the model [70].

Maintaining both transferability and performance is a challenge when constructing any radiomics model. Transferability can be achieved in some capacity by restricting a model to features that are stable when acquired with different parameters [36]. It can be difficult to maintain performance of a model while restricted to these features. Additionally, difficulties can arise in radiomics due to the lack of confidence around the reproducibility of radiomic features. A lack of reproducibility in features will mean that radiomic models will be inaccurate in creating predictions [32].

Challenges arise in constructing radiomics models for brain cancer in establishing a biological ‘ground truth’ [25]. As radiomics models are often designed to predict biological characteristics of tissue, it is necessary to obtain the biological data for training these models. A biopsy is considered the gold standard for assessing biological function of tissue. However, using a biopsy in conjunction with radiomics is non-trivial for brain cancer. Challenges include that whole brain resection cannot be performed to investigate the biology of the whole brain; accurate image-guided biopsies are difficult to perform due to brain shifts on craniotomy [71]; challenging tumour location in critical areas of the brain prevent the performance of biopsies; and fragility of brain tissue compromises the retention of the tissue structural integrity. These factors make it difficult to assess the accuracy of radiomics models via image-guided biopsy. This creates challenges in assessing intratumoural heterogeneity, due to the difficulty in identifying biopsy location and retaining spatial information of the sampled tumour tissue. A study aiming to achieve spatial accuracy in GBM biopsy had a mean error of 1.5 ± 1.1 mm; however, this required MRI to be completed during the biopsy procedure [71]. Challenges in retaining spatial registration between the biopsy location and the region of interest marked on the image make it difficult to build radiomic models predicting biological heterogeneity with high accuracy. The alternative to biopsy is for an expert radiologist to label a tumour based on available imaging; however, this method may suffer from poor accuracy in comparison to biopsy.

GBM also poses the challenge of having tumour infiltration that extends beyond the visible margins of contrast enhancement. Tumour infiltration is not visible on conventional MRI due to the presence of an intact BBB preventing the contrast agent from entering the tumour extracellular extravascular space. As such, accurate delineation of the tumour boundaries beyond the extent of contrast enhancement on T1CE images is extremely challenging, and clinicians are forced to rely on margins of uncertainty in planning treatments, which ultimately contributes to suboptimal treatment efficacy [72]. This presents both a challenge and an opportunity for radiomic research. Radiomic research can suffer from not having the full extent of the tumour delineated for feature extraction; however, it has also shown some promise in identifying regions of peritumoural infiltration by integrating machine learning methods into the analysis pipeline [12].

## 5. Improvements Required to Build Confidence in GBM Radiomics

### 5.1. Multi-Parametric Radiomics Models

Advanced imaging techniques, including multiparametric MRI, and advanced analysis methods, including ML algorithms, have led to improvement in the performance of radiomics models.

To date, research in GBM radiomics has primarily utilised anatomical (T1, T2, FLAIR) MRI sequences. However, much stands to be gained in terms of model performance by implementing functional imaging sequences into radiomics. Given that radiomics aims to predict clinical outcomes of patients based on imaging features, which are related to underlying tissue physiology, it stands to reason that images quantifying tissue physiology should contain valuable information for radiomics. Specialised MRI sequences that have shown promise in GBM radiomics include [10]:

- Diffusion weighted imaging (DWI)—MRI sequences which quantify diffusion of water molecules [19];
- Perfusion weighted imaging (PWI)—Group of MRI sequences which quantify perfusion parameters [19];
- Magnetic Resonance Spectroscopy (MRS)—MRI spectroscopy sequence to identify the presence of certain metabolites [73].

In addition to MRI, PET imaging has shown some utility in performing a functional assessment of GBM [26,27]. PET imaging in GBM has most often utilised Fluoro-deoxy Glucose (FDG-PET) [74] as a marker of metabolism or flouro-L-tyrosine (FET-PET) as a marker of protein synthesis [26].

### 5.2. A Roadmap for the Implementation of Radiomics in Clinical Practice

The development pathway for radiomics models can broadly be broken down into three main steps, discovery, validation and clinical implementation [24,28]. During the discovery stage, radiomic signatures are selected to be predictive of clinical or biological parameters within existing data. During the validation stage, models are tested on diverse data. During clinical implementation and validation, models passing the discovery stages are implemented into clinical trials to assess their performance within a clinical setting. For now, the development of radiomics models largely remains in the discovery stage.

Within GBM, it is important to develop models designed to predict clinically useful parameters. Within GBM patient management, this means measuring parameters useful for treatment adaptation to improve patient outcomes. This is the primary purpose of the discovery stage of radiomics and research has found some useful correlations for the prediction of biological and clinical parameters. Useful predictions for implementation of radiomics into GBM patient management include: stratification of patients to determine if they will respond to a certain therapy [62]; early assessment of treatment response to assess the need for treatment adaptation [75]; prediction of patient survival [19] or recurrence and determination of peritumoural infiltration [12]. Each of these tasks can be performed by radiomics and provides valuable information for treatment adaptation.

Barriers to clinical implementation of radiomic models arise from the lack of transferability in the development [25]. Standardisation of imaging acquisition protocols and development of image analysis standardisation methods are necessary for the development of transferable radiomics model [10,76]. It will also be necessary to standardise the feature extraction and analysis methods used to develop radiomics models. Improving the public availability of imaging datasets with supporting data, such as clinical outcomes or biopsy results, will be beneficial as uncertainties in ML techniques are linked to the small sample sizes in training sets. Improved availability of data from multiple sites will also allow for the development and validation of radiomics models on multicentre data. This, in turn, will enable the assessment of the transferability of radiomics models. The existence of publicly available multicentre data through the Cancer Imaging Archive (TCIA) [77] and the BraTS challenge [11] has helped in this regard with datasets from these sources being used in multicentre validation studies [36,68].

One issue facing the development of radiomics models for GBM is that many of the modern models are developed using the same datasets, those being the TCIA [77] datasets and the BraTS [11,45,57] dataset. These datasets include patients treated before 2000, potential segmentation errors and images acquired with widely varying protocols and image quality. Despite these datasets making up a large amount of the data being analysed in the radiomics space, there has not been extensive work into assessing the quality of these data [77]. Without a complete quality assessment, the accuracy of the predictions can not be assured.

It is important for models to improve their performance. With the performance achieved by the modern radiomics models referenced in Table 2, it is unlikely that these will be considered for implementation in clinical practice. The AUC is a common metric used to assess the performance of binary prediction models which ranges from 0.5–1 with 0.5 being no better than random and 1 being a perfect prediction. Generally, a value greater than 0.8 is considered a good prediction while above 0.9 is excellent. There is not an agreed minimum level of performance for implementation in clinical practice. The acceptable level of performance will need to be decided by clinicians depending on the task that a radiomics model is performing [78]. It is also important that the acceptable level of performance is compared to what is currently achieved in the clinic, although determining these values can also be a major area of research. This level of performance may be achieved by using high quality, multi-parametric data in training and implementing advanced analysis methods to build these models.

Clinical trials evaluating the effects of the using radiomics models on patient treatment outcomes will be necessary to see widespread clinical implementation of radiomics. However, before such studies can be performed, standardised imaging and analysis protocols ensuring reliability of models predictions should be established [28].

Another important consideration for the development of radiomics models towards clinical implementation is the knowledge gap between data experts and clinicians. While radiomics models have the potential to provide useful information to clinicians, it is important that clinicians understand how this information is developed and what its limitations might be. Conversely, it is also important for data experts who develop these models to have an understanding of what important and relevant endpoints a radiomics model should predict or include and what performance a model should have to be clinically useful.

## 6. Conclusions

The development of radiomics models aimed to assist in the management of GBM in recent years has yielded promising results. The increased use of ML methods, and multi-modal imaging has driven an improvement in performance of GBM radiomic models. To overcome the challenges associated with GBM radiomics, increased access to diverse, large datasets will be necessary. In pursuit of this goal, multicentre collaborations should be sought. Further development in this direction should improve the performance, usefulness and transferability of emerging radiomics models, bringing them one step closer to clinical implementation.

## Figures and Tables

**Table 1:** A summary of ML algorithms used in radiomics research.

Algorithm	Description	Application in Radiomics
Support Vector Machine	A support vector machine aims to perform binary classification on multidimensional data by finding the ideal hyperplane to separate the two classifications	Support vector machines can be used on a voxel-by-voxel basis to predict tissue biological parameters or in conjunction with radiomic features to make a binary prediction (for example distinguishing between high and low grade glioma) based on multiple feature values. This was implemented in a study by Qian et al. [43] to differentiate between Glioblastoma and Gliosarcoma.
Neural Network	A neural network performs mathematical operations on input data through a series of interconnected layers to produce a prediction. Deep Learning is a subset of Machine Learning based on neural networks using two or more ‘hidden layers’ and has received much attention in recent years for image and data processing.	Neural networks can be used in place of a regression algorithm to generate predictions based on the values of radiomic features [12]. In the case of deep learning, a class of radiomic features known as deep features that are derived using convolutional neural networks. In addition to this, deep learning has been implemented in automated segmentation of brain tumours [44,45].
Random Forest	A random forest is an ensemble of decision trees with a final prediction created by the results of all the trees. The final decision is created by a ‘vote’ of all these trees.	A model aiming to produce a binary prediction based on multiple factors could benefit from the implementation of random forests. Tasks related to GBM patient management suited for including differentiation between pseudo- and true- tumour progression, stratification of patients into high or low risk categories [46] and a grading of gliomas [47].

**Table 2:** A selection of modern radiomics models for predicting clinical and biological factors in GBM.

Author	Model Description	Conclusions	Clinical Application	Performance	Patient Numbers
Kickingereder et al., 2016 [62]	Stratification of patients into groups who were likely or not likely to benefit from anti-angiogenic therapies	A radiomics model based on supervised principal component analysis is effective at stratifying patients into groups that can benefit from the addition of anti-angiogenic therapy	Identification of which patients may benefit from certain therapies provides clinicians a convenient to tailor treatment regimens to individuals	AUC = 0.792	172
Lao et al., 2017 [38]	Deep features were extracted using transfer learning and implemented into a survival prediction model. This model utilised learned features, handcrafted radiomics features and clinical factors to produce a prediction of overall patient survival.	Implementing learned features into a predictive radiomics model can improve the performance of a predictive model.	A survival prediction model can be used to determine if a patient would benefit from a more aggressive treatment regimen. Improving performance by implementing learned features and clinical factors can build confidence in the model.	AUC = 0.739	112
Shboul et al., 2019 [23]	A fully automated segmentation pipeline using Deep Neural Networks was developed using the BraTS challenge dataset. Survival prediction was then performed using radiomic features extracted from this dataset.	A fully automated framework for the delineation of GBM and patient survival prediction can be useful to reduce clinical workload and bias in the tasks of segmentation and survival prediction.	A framework such as this can be used to provide a perform a tumour segmentation for the purpose of radiotherapy treatment planning. Survival predictions can be used to recommend a more or less aggressive treatment regimen as required	Leave one out cross validation accuracy = 0.73	396 total
Park et al., 2020 [19]	Survival Prediction based on T1 Post Contrast, T2 FLAIR and DSC MRI as well as clinical factors.	By incorporating mpMRI as well as clinical factors, it is possible to achieve a high performing survival prediction model	An accurate prediction of survival period can provide a quantitative measure of the severity of the disease.	AUC = 0.74	216
Yan et al., 2020 [12]	Identification of peritumoural invasive regions in GBM based on Structural, Perfusion-weighted and Diffusion-weighted MRI. Convolutional Neural Network was used along with radiomics to identify regions of peritumoural infiltration	Lower intensity on Diffusion-weighted MRI and higher intensity on T1, FLAIR and Perfusion-weighted MRI was observed in peritumoural invasion areas.	Identification of regions of peritumoural invasion will allow treatment plans to accurately target whole tumour volumes and improve local control.	Accuracy = 78.5%	57
Suter et al., 2020 [36]	Feature robustness was tested and models developed on single centre data were applied to multicentre data. In addition to this, a model developed using robust features on single centre data was tested on multicentre data.	A large performance drop was found when models trained on single centre data were applied to multicentre data. This performance drop could be reduced when the model was restricted to robust features.	Model transferability is an important factor in radiomic research. To develop transferable radiomics models, it will be necessary to develop models on multi-centre data and identify reproducible radiomic features.	AUC reduced by 0.56 for single centre model tested on multicentre data	63 single centre patients, 76 multicentre data
Shim et al., 2021 [22]	Prediction of recurrence pattern based on DSC MRI radiomics and neural networks, model produced to predict local and distant recurrence	Quantitative measures of tumour perfusion can accurately predict recurrence patterns of tumour recurrence	Identifying the likely course of tumour progression could enable early intervention or treatment plan adaptation.	AUC = 0.969; AUC = 0.864 (local and distant)	192

DSC = Dynamic Susceptibility Contrast, mpMRI = multi-parametric MRI.
